# Confinement and Exciton Binding Energy Effects on Hot Carrier Cooling in Lead Halide Perovskite Nanomaterials

**DOI:** 10.1021/acsnano.2c12373

**Published:** 2023-03-20

**Authors:** Ben P. Carwithen, Thomas R. Hopper, Ziyuan Ge, Navendu Mondal, Tong Wang, Rozana Mazlumian, Xijia Zheng, Franziska Krieg, Federico Montanarella, Georgian Nedelcu, Martin Kroll, Miguel Albaladejo Siguan, Jarvist M. Frost, Karl Leo, Yana Vaynzof, Maryna I. Bodnarchuk, Maksym V. Kovalenko, Artem A. Bakulin

**Affiliations:** † Department of Chemistry and Centre for Processable Electronics, Imperial College London, London W12 0BZ, United Kingdom; ‡ Department of Materials Science and Engineering, Stanford University, Stanford, California 94305, United States; § Laboratory of Inorganic Chemistry, Department of Chemistry and Applied Biosciences, ETH Zürich, CH-8093 Zürich, Switzerland; ∥ Laboratory for Thin Films and Photovoltaics, Empa−Swiss Federal Laboratories for Materials Science and Technology, CH-8600 Dübendorf, Switzerland; ⊥ Zernike Institute for Advanced Materials, University of Groningen, Nijenborgh 4, Groningen 9747AG, The Netherlands; # Center for Advancing Electronics Dresden, Technische Universität Dresden, 01069 Dresden, Germany; ∇ Integrated Center for Applied Photophysics and Photonic Materials, Technische Universität Dresden, 01187 Dresden, Germany; ○ Chair for Emerging Electronic Technologies, Technische Universität Dresden, 01187 Dresden, Germany; □ Leibniz Institute for Solid State and Materials Research Dresden, Technische Universität Dresden, 01069 Dresden, Germany

**Keywords:** hot carriers, two-dimensional perovskites, nanoplatelets, nanocrystals, ultrafast spectroscopy

## Abstract

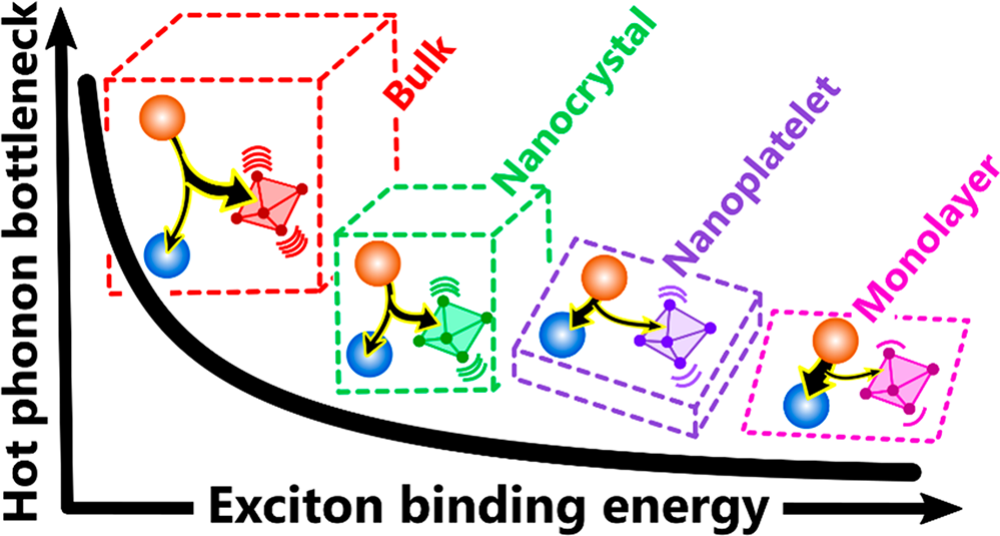

The relaxation of the above-gap (“hot”) carriers in lead halide perovskites (LHPs) is important for applications in photovoltaics and offers insights into carrier–carrier and carrier–phonon interactions. However, the role of quantum confinement in the hot carrier dynamics of nanosystems is still disputed. Here, we devise a single approach, ultrafast pump–push–probe spectroscopy, to study carrier cooling in six different size-controlled LHP nanomaterials. In cuboidal nanocrystals, we observe only a weak size effect on the cooling dynamics. In contrast, two-dimensional systems show suppression of the hot phonon bottleneck effect common in bulk perovskites. The proposed kinetic model describes the intrinsic and density-dependent cooling times accurately in all studied perovskite systems using only carrier–carrier, carrier–phonon, and excitonic coupling constants. This highlights the impact of exciton formation on carrier cooling and promotes dimensional confinement as a tool for engineering carrier–phonon and carrier–carrier interactions in LHP optoelectronic materials.

In the past decade, lead halide perovskites (LHPs) were introduced as promising systems for optoelectronic applications.^1^ LHP solar cells have seen a rapid increase in power conversion efficiencies^2−4^ and are fast-approaching the theoretical limit for single-junction devices. LHP-based light-emitting diodes have also shown promising performance metrics, including color purity and stability.^5^

LHP materials offer broad tunability of their optoelectronic properties through varying the composition, size and dimensionality of bulk and nanoscale systems.^6^ All of these material parameters influence the energy and localization of electronic states, density and distribution of phonon modes, and electron–phonon coupling. This allowed the development of materials with specific absorption, emission, and charge transport properties suitable for applications in a wide range of optoelectronic devices.

An important aspect of the photophysics of bulk and confined semiconductors is the ultrafast relaxation of “hot” carriers following above-gap optical or electronic excitation.^7−10^ Carrier cooling in LHPs initially attracted attention for hot carrier solar cells^11,12^ and light-emitting applications,^13^ but focus has now extended toward more fundamental studies concerning carrier–carrier and carrier–phonon coupling and their interplay. Further insight into the photophysics of these materials may be found through the systematic study of cooling dynamics in different types of LHP.

Hot carrier cooling dynamics in bulk and nanocrystalline halide perovskites have typically been studied using time-resolved photoluminescence (PL)^14−17^ or broadband transient absorption spectroscopy (TAS), with a few other notable exceptions.^18−23^ In TAS studies, cooling rates can be determined after above-gap excitation from (a) extraction of an effective carrier temperature from the high-energy tail of the ground state bleach (GSB);^24−30^ (b) the delayed onset of the GSB formation due to state filling;^31^ (c) the decay of a subgap photoinduced absorption arising from the Stark effect; or a combination thereof.^32,33^ As such, sophisticated analyses are required to account for the various overlapping spectral responses.^34^ Furthermore, before pseudoequilibrium of carriers has been established following photoexcitation, and in excitonic systems with quantized states, the definition of electronic temperature is somewhat unclear.^35^

The general picture of hot carrier cooling that has emerged from these studies consists of a sequence with three stages: (i) Pseudoinstantaneous photoexcitation produces a nonthermal distribution of carriers; (ii) Within 100 fs, thermalization via elastic scattering results in a Fermi–Dirac distribution of carriers with an effective temperature greater than that of the surrounding lattice;^30,36,37^ (iii) Cooling of this distribution via deposition of excess carrier energy into longitudinal-optical (LO) phonons^38^ results in band-edge (“cold”) carriers within ∼1 ps. There are also some reports of longer-lived cooling components, possibly due to acoustic phonon scattering^20,39^ or Auger reheating.^25^

For most systems, cooling has been shown to slow at a higher carrier density. This phenomenon is often ascribed to the hot phonon bottleneck, where the increased competition for a finite number of available phonon modes results in the continuous emission and reabsorption of hot phonons by carriers.^24,40−43^ This is more pronounced in LHPs compared with other classes of semiconductors, which has been variously attributed to polaron screening,^14^ inefficient Klemens decay of optical phonons,^25^ and acoustic–optical phonon upconversion.^44,45^

In quantum-confined systems, electronic band continua are expected to acquire fine structure and even be segregated into discrete states. This ought to slow hot carrier cooling rates if the separation between states is greater than the energy of the coupled LO phonons.^46^ However, cooling was instead shown to be faster in CdSe^47−50^ and PbSe^51,52^ nanocrystals (NCs) than in their bulk analogues, owing to the dominance of other channels such as electron–hole scattering.^9,53^

More recently, with the advent of facile synthesis routes to perovskite nanomaterials,^54−59^ attention has been turned to carrier cooling in these systems.^33,60^ While there are some reports of slowed cooling in cuboidal perovskite NCs,^61^ many other studies have revealed cooling behavior that is similar to bulk systems.^26,32,62−65^ LHP nanoplatelets (NPLs) are anisotropic variants of NCs, i.e., are more strongly confined in one dimension than the other two, and have displayed faster cooling than their bulk counterparts,^66,67^ while contrasting cooling behavior in single-monolayer quantum wells has been reported to be either faster^68,69^ or slower^45,70^ than the bulk. Some reports have also shown slowed carrier cooling through crystal alignment engineering.^71,72^ The recurring theme in these works is a modulation of the exciton–phonon coupling strength in two-dimensional (2D) perovskites through a reduction in carrier screening,^73^ partly due to the increased dielectric-confinement imparted by the organic spacer ligands.^45,67,74−77^

Pump–push–probe (PPP) spectroscopy has been introduced as a means of isolating intraband relaxation in systems ranging from inorganic NCs^7,52,78−80^ to molecular systems.^81^ We,^40,41,82^ and others,^83−86^ have recently employed PPP spectroscopy to complement existing TAS studies of bulk and nanocrystalline LHPs. This three-pulse technique has yielded results qualitatively similar to TAS studies, e.g., the presence of a hot phonon bottleneck at a high hot carrier density. However, PPP offers additional control over the relative hot and cold carrier subpopulations, which allows us to identify and separate cooling pathways mediated by carrier–phonon and carrier–carrier scattering. Application of the PPP technique to a large ∼8 nm (cf. Bohr exciton diameter, *d*_B_ ∼ 7 nm^55^) CsPbBr_3_ NC showed no differences in cooling behavior with respect to their bulk counterparts, suggesting that the electronic state and phonon distributions are not significantly perturbed by quantum confinement in these systems, nor conspicuously affected by surface trap states.^41^

Here, hot carrier cooling dynamics are investigated in a range of perovskite nanomaterial architectures: 5 nm cuboidal CsPbBr_3_ NCs, 2D CsPbBr_3_ NPLs (∼3.5 × 11 × 14 nm), and Ruddlesden–Popper (RP)-type (PEA)_2_PbI_4_ and (PEA)_2_PbBr_4_ (PEA = phenylethylammonium). Through these comparisons, we reveal the effect of confinement on hot carrier cooling, allowing for the factors governing carrier–phonon and carrier–carrier coupling in perovskites to be inferred. We observe a weak dependence of size on cooling dynamics in cuboidal NCs, while few-monolayer NPLs and RP-type perovskites show a suppressed hot phonon bottleneck effect, which we attribute to increased carrier–carrier interactions inherent to these systems.

## Results and Discussion

Ultrafast PPP spectroscopy was implemented to isolate the hot carrier cooling dynamics in these systems. Here, a 490 nm “pump” excites carriers across the band gap, and their time-evolving population is monitored by the absorption of a low intensity 2 μm “probe” corresponding to an intraband transition in the broad excited state spectra of LHPs in the near-infrared (NIR).^87,88^ Owing to their similar effective masses in halide perovskites,^89^ electrons and holes are not discriminated in this work, and the discussion of cooling dynamics herein is generalized to both types of charge carrier.

**Figure 1:**
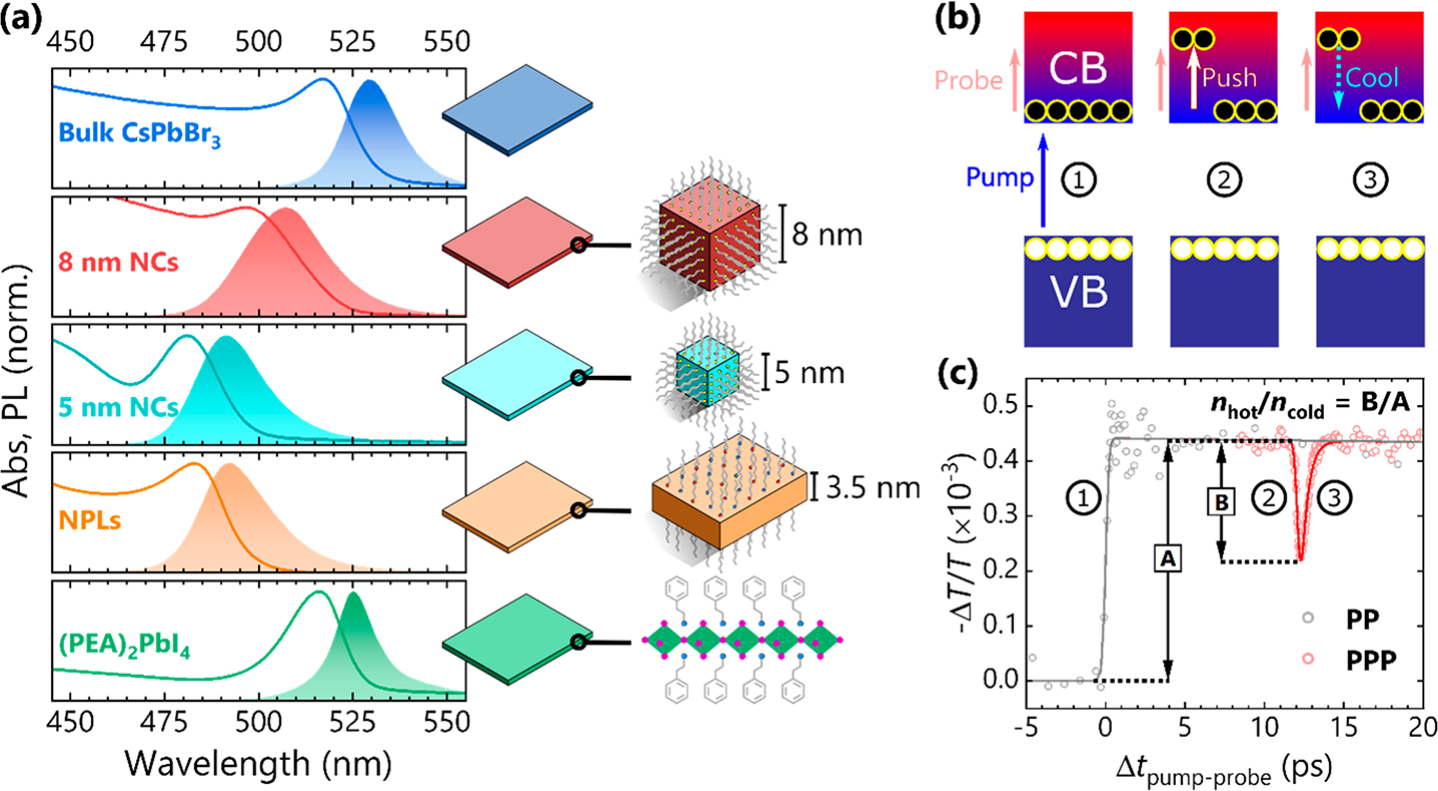
(a) Absorption (solid lines) and PL (shaded) spectra for the studied LHP nanomaterials, and bulk and 8 nm “large” NC references. (b) Action of pump, push and probe on carriers, and (c) their resulting effects on exemplar PP and PPP transients for 5 nm CsPbBr_3_ NCs; *n*_hot_/*n*_cold_ is calculated from the ratio of the push-induced bleach magnitude (B) to the background PP signal magnitude (A). Further details can be found in the main text.

At a fixed delay (∼12 ps) after the pump, an intense 2 μm “push” pulse impinges on the excited region of the sample, optically re-exciting the carriers from the band-edge to a hot state and bleaching the probe absorption (Figure 1c). That is, using this narrowband NIR PPP scheme we reduce the system to a two-state (hot or cold) carrier model and avoid the potential issues with extracting a carrier temperature from a broadband probe described earlier. Instead, the probe response is proportional to the population of cold carriers and therefore so too is the push-induced bleach. With increasing push fluence, a higher density of hot carriers is produced with a correspondingly greater bleach magnitude. Fitting the bleach and its subsequent recovery with a Gaussian-modified exponential function yields a time constant associated with cooling. The width of the Gaussian instrument response function was 100 fs for all data sets (see also Figure S1).

By employing this double-excitation scheme, independent control of the cold and hot carrier densities (via pump and push fluence, respectively) is achieved, and their effects on carrier cooling can be discerned. Importantly, the same pump wavelength may be used in PPP experiments irrespective of the band gap of the material under study provided *hc*/λ_pump_ ≥ *E*_g_. That is, regardless of the initial excess energy of carriers formed by the pump, the delayed push interacts with a purely band-edge (cold) carrier distribution, and any pump-induced reorganization processes will be complete prior to the push arrival.^23^ Using a consistent push wavelength ensures hot carriers are (re-)formed with the same excess energy across all systems. It should also be noted that the push does not induce thermal population changes because the time scales of the probe bleach and recovery are too short to allow thermal equilibrium of carriers and the lattice to be attained.^90^ Moreover, while the laser pulse energy would eventually be deposited into the lattice, the typical thermal transport time scales in these materials are much shorter than the 250 μs (*f* = 4 kHz) pulse separation. Thus, thermal effects should not “build up” over the course of a measurement.

All materials were fabricated via previously reported procedures,^54,58,91^ which are detailed in full in the Methods section and SI, Section A. This yielded CsPbBr_3_ NCs with a side length of 5 nm, capped with the zwitterionic 3-*N*,*N*-(dimethyloctadecylammonio)propansulfonate ligand; and CsPbBr_3_ NPLs with a thickness of ∼3.5 nm (or 6 monolayers) and lateral dimensions of ∼11–14 nm, passivated by a mixture of oleate and oleylammonium ligands. The dimensions of the nanosystems were confirmed by absorption and PL spectra (Figure 1a) and electron microscopy images (Figure S2).^54^ Their optical spectra are blue-shifted with respect to their larger analogues and also exhibit additional excitonic transitions at energies above the absorption onset (Figure S3), corroborating the effects of quantum confinement on their band structure. The even smaller “critical thickness” in the NPLs causes a wider band gap and greater energy difference between the lowest-lying excitonic states than in the NCs.

Drop- or zone-casting from a solution of (PEA)_2_PbI_4_ in dimethylformamide (DMF) resulted in films of 25 and 6 μm thickness, respectively (Figures S4 and S5). (PEA)_2_PbBr_4_ films were also drop-cast from DMF. The extent of blue-shifting and exciton peak separation in the RP-types is more pronounced than in the CsPbBr_3_ nanomaterials, pointing to the greater excitonic character of excited state species in the former.^91^

**Figure 2:**
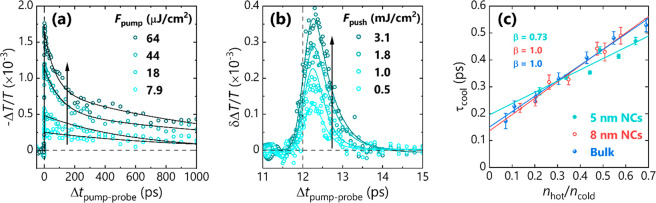
(a) Pump fluence-dependent kinetics of the intraband carrier absorption in 5 nm CsPbBr_3_ NCs. (b) Push fluence-dependent kinetics of the push-induced bleach of the intraband absorption at a fixed pump fluence of 10 μJ cm^–2^. (c) Size-dependence of hot carrier cooling dynamics in bulk and NC CsPbBr_3_. β values denote slope of fitted lines (see main text for details).

Figure 2a shows the pump fluence-dependent pump–probe (PP) transients for CsPbBr_3_ NCs with ∼5 nm edge length. At low pump fluence and therefore low initial carrier density, the main decay pathway is electron–hole recombination, and the dynamics can be described by a single component with a time constant of ≫1 ns, consistent with time-resolved PL studies.^55^ At high carrier density, Auger-type recombination is observed as a second, shorter decay with <100 ps lifetime. The overall behavior of the carriers across these excitation regimes, shown here through intraband absorption of a narrowband NIR probe, is congruent with findings from earlier broadband TAS studies.^64^

Figure 2b and Figure S6 show the push fluence-dependent bleach in PPP transients for 5 nm NCs. For all PPP experiments, the pump was kept at a fluence that produced a sufficiently low photocarrier density to remove any contribution from Auger-type recombination processes, thereby maximizing signatures of carrier–phonon and carrier–carrier interactions that determine the strength of the hot phonon bottleneck effect. We note that the two-body carrier–carrier scattering process will still be present under pump fluences that preclude three-body Auger processes, which involve the transfer of energy from electron–hole recombination to a nearby third carrier. The push-induced bleach returns to the PP baseline for all PPP transients of all studied systems, implying that neither multiphoton push absorption^92−94^ nor trapping (e.g., due to surface ligands as discussed later) impact the observed dynamics.

To investigate the variations in cooling times and hot phonon bottleneck behavior in these systems, the cooling time constant is plotted as a function of hot carrier density (*n*_hot_) immediately following push excitation, normalized to the cold carrier density (*n*_cold_) just before the push (*n*_hot_/*n*_cold_, Figure 1c). This metric is advantageous because it (i) eliminates the need to calculate exact carrier densities, which would otherwise require an accurate measurement of the pump and push/probe absorption cross sections across different areas of the sample; and (ii) accounts for the effects of both *n*_hot_ and *n*_cold_ on cooling dynamics, such that different materials may be compared, assuming *n*_cold_ is not too high.^95^ Further validation of this metric for nanocrystals is given via a Monte Carlo simulation of the hot phonon bottleneck effect, outlined in SI Section B. As we have shown previously,^40,41^ there is a linear increase in cooling time with increasing hot carrier density for bulk thin-film and large NC perovskite analogues. A steeper slope indicates a more pronounced hot phonon bottleneck and is a marker of reduced underlying carrier–phonon interactions.^40^ An additional cooling pathway mediated by hot–cold carrier scattering exists wherein at higher *cold* carrier densities, hot carriers can more efficiently deposit their energy into the reservoir of cold states.^41^ Hereinafter, a parameter β is used to indicate the strength of the hot phonon bottleneck for each system and is calculated from the slope of τ_cool_ with respect to *n*_hot_/*n*_cold_ for each material, normalized to the slope for bulk CsPbBr_3_. The *y*-intercept of these plots can be attributed to the intrinsic cooling time of an isolated hot carrier.

As previously reported,^41^ 8 nm CsPbBr_3_ NCs showed identical cooling behavior to their bulk analogue despite quantum confinement being evident from their blue-shifted absorption and emission spectra. Figure 2c shows the cooling behavior of moderately confined 5 nm CsPbBr_3_ NCs alongside the 8 nm NCs and bulk thin-film analogues. Compared to the larger NCs with the same ligands, the smaller NCs show broadly similar cooling behavior but with a slightly flatter slope (β = 0.73 ± 0.07), i.e., a suppressed hot phonon bottleneck effect. This agrees with reports introduced earlier, wherein small differences in cooling dynamics were observed for NCs with edge lengths greater than ∼4 nm.^26,62,63^ We attribute this to the size of the crystals not further confining the charge carriers below the 2.5 nm polaron radius predicted from Feynman–Schultz theory.^96^

**Figure 3:**
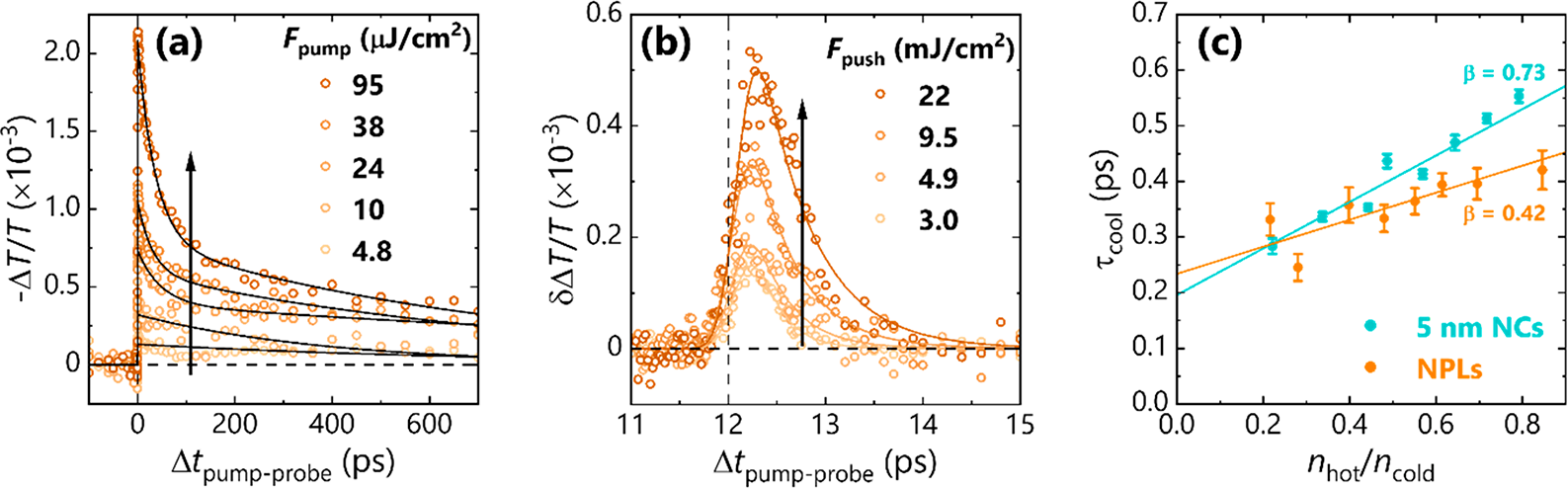
(a) Pump fluence-dependent kinetics of the intraband carrier absorption in CsPbBr_3_ NPLs. (b) Push fluence-dependent kinetics of the push-induced bleach of the intraband absorption, at a fixed pump fluence of 10 μJ cm^–2^. (c) Dimensionality-dependent hot carrier cooling dynamics in CsPbBr_3_ nanomaterials. β values denote slope of fitted lines (see main text for details).

Figure 3a shows the PP carrier dynamics for 3.5 nm-thick CsPbBr_3_ NPLs under varying pump fluence. At low fluence, a single exponential decay component is observed with a ∼1 ns lifetime, representing electron–hole band-edge recombination. At higher fluence, a faster ∼10 ps decay pathway emerges which is ascribed to Auger-type recombination.^97^

Figure 3b shows the push fluence-dependent bleach recovery in the NPLs, from which the cooling dynamics as a function of *n*_hot_/*n*_cold_ are derived in Figure 3c. The NPLs are more strongly confined in one dimension than the cuboidal 5 nm NCs, but less so in the lateral dimensions. While the passivating ligands used in the NCs and NPLs also differ,^62^ previous studies have shown that the dimensionality of the LHP lattice has a much greater impact on electronic properties^98^ and cooling dynamics^35,41,62^ than surface chemistry. When compared to 5 nm NCs, the NPLs display a suppressed hot phonon bottleneck (β = 0.42 ± 0.08). While this may suggest that there are more (or better-coupled) accessible phonon modes in the NPLs, other studies revealed negligible changes in the phonon spectra^99,100^ and Fröhlich coupling constant^73^ in systems of the same thickness as described in this work. A more likely explanation for the suppressed hot phonon bottleneck is, therefore, the enhanced influence of carrier–carrier scattering, involving the interactions between an electron and hole bound in the same exciton.^47,48,80^ In other words, the presence of a cooling pathway mediated by carrier–carrier scattering increases the critical hot phonon density required to slow carrier cooling, which flattens the observed dependence of τ_cool_ on *n*_hot_. Herz et al.^83^ also recently highlighted the role of cold carriers in the hot carrier dynamics of Sn-based perovskites using pump–push–THz-probe spectroscopy, and Ruhman et al.^78^ discuss the effect of cold spectator excitons in the intraband relaxation of PbS NCs.

To investigate whether the effects observed so far are specific to NPLs or generally applicable to 2D perovskites, where excitonic character is enhanced further, we extended our measurements to RP-type (PEA)_2_PbI_4_. This excitonic enhancement in 2D materials is well understood as being due to reduced screening and therefore greatly enhanced Coulomb interactions between electrons and holes. As such, this allows us to comment further on the influence of confinement on hot carrier cooling dynamics.

**Figure 4:**
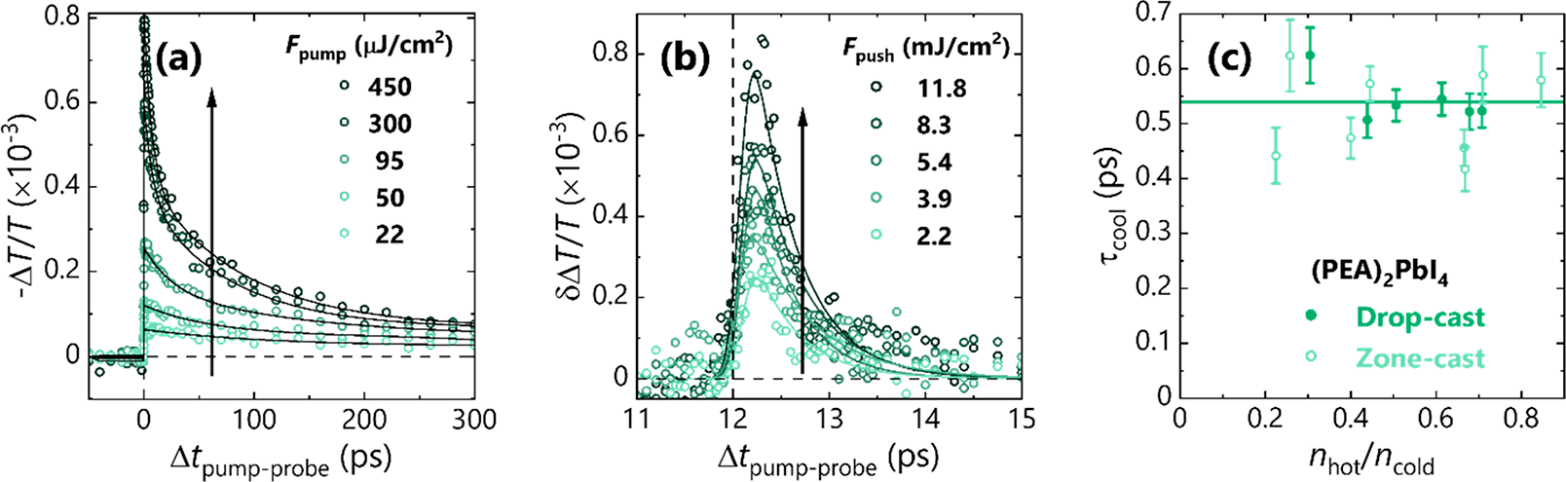
(a) Pump fluence-dependent kinetics of the intraband carrier absorption in zone-cast (PEA)_2_PbI_4_. (b) Push fluence-dependent kinetics of the push-induced bleach of the intraband absorption, at a fixed pump fluence of 30 μJ cm^–2^. (c) Completely suppressed hot phonon bottleneck in (PEA)_2_PbI_4_ for both film types; the line shows the average of extracted cooling times.

The PP transients for (PEA)_2_PbI_4_ (Figure 4a) behave similarly to the previously discussed nanosystems, in that the dynamics show evidence of nonlinear decay pathways with increasing pump fluence. However, owing to the higher exciton binding energy here, the predominant species is assumed to be interacting charge pairs (excitons) rather than free carriers. At low fluence, a single decay component with ≫100 ps lifetime is observed, which is assigned to radiative exciton recombination.^101^ This is shorter than in the NCs and NPLs described above, which is common for low-dimensional systems due to their greater oscillator strength.^75,102^

Figure 4b,c and Figures S7 and S8 show the cooling behavior found in (PEA)_2_PbI_4_ thin-films. There is no correlation between *n*_hot_ and τ_cool_, which implies the lack of a hot phonon bottleneck in these materials (β < 0.2). However, the cooling times of ∼500 fs are consistently longer than in all other materials studied. This is not surprising considering the very different nature of the electronic states, phonon spectrum, and electron–phonon coupling in this system as compared to that of (nanoconfined) ABX_3_ perovskites. This behavior is identical in both drop- and zone-cast films despite their different grain sizes and alignment (Figures S4 and S5). Therefore, any effects from intergrain coupling are not manifested in the hot carrier cooling dynamics, which further supports the notion that the surface states and ligands are decoupled from the cooling dynamics. The complete suppression of the hot phonon bottleneck in (PEA)_2_PbI_4_ is ascribed to the enhanced electron–hole coupling in this excitonic system, arising from the reduction in screening and resulting stronger Coulomb interactions. This influence of the electron–hole interaction on the carrier dynamics was previously observed in PbS nanocrystals.^66^

We have shown previously that the intrinsic hot carrier lifetime in iodide-based perovskites is longer than that of their bromide analogues,^40^ owing to the weaker electron–phonon coupling in the former.^103^ This partially explains the difference in behavior between (PEA)_2_PbI_4_ and CsPbBr_3_ nanomaterials. However, such a stark difference may additionally arise from the strongly excitonic character of (PEA)_2_PbI_4_: if the electronic density of states in this material consists of quasi-discrete regions, carrier cooling ought to be intrinsically slower (higher intercept). Within this same framework, cooling dominated by carrier–carrier scattering would produce a suppressed hot phonon bottleneck (flatter slope). The greater influence of the organic spacer ligand in quantum wells than in the larger systems is also expected to impact carrier dynamics.^66^

We note that the absence of a hot phonon bottleneck and a slower intrinsic hot carrier lifetime were also observed for the bromide RP-type (PEA)_2_PbBr_4_ (Figure S9c). However, the substantially wider electronic band gap (Figure S9a) and the shorter exciton lifetime seen in PP experiments (Figure S9b) made probing the hot carrier dynamics by the delayed push pulse challenging. Hence, the main discussion has been presented on (PEA)_2_PbI_4_ which has optoelectronic parameters comparable to those of the other studied materials.

Cassette et al.^35^ recently observed an enhanced hot phonon bottleneck in RP-type perovskite films with respect to colloidal NPL systems of the same number of monolayers, albeit using higher pump fluences than in the present study; while Wang et al.^104^ observed the absence of a hot phonon bottleneck in isolated CsPbBr_3_ quantum wells, which became activated upon the formation of a layer-stacked superlattice. Both these studies are consistent with our findings that demonstrate a suppression of the hot phonon bottleneck in more strongly confined LHP nanomaterials. We note that in analogous studies on PbS and PbSe quantum dots^105,106^ the hot phonon bottleneck was instead stronger for a greater interdot spacing due to the decoupling of carriers and phonons.

**Figure 5:**
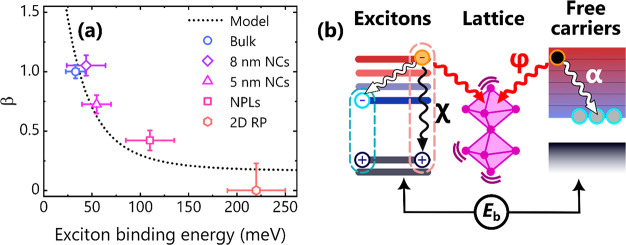
(a) Hot phonon bottleneck parameters (β) for all CsPbBr_3_ systems and (PEA)_2_PbI_4_. A larger β value implies a more pronounced hot phonon bottleneck. Error bars denote the 99% confidence interval. The black dotted curve is the hot phonon bottleneck behavior as a function of *E*_b_ predicted by the kinetic model described in the main text and panel (b) where α, ϕ, and χ are the hot–cold carrier, carrier–phonon, and hot exciton cooling coefficients, respectively.

As summarized in Figure 5a, there is a clear trend across the studied systems: with increasing exciton binding energy (*E*_b_), the hot phonon bottleneck becomes more suppressed. *E*_b_ values were obtained by modeling the absorption spectra using Elliott theory^107−109^ and are consistent with those reported elsewhere (see SI Section C and Figure S3).^77,110−112^ The partitioning of total hot states into hot excitons (*n*_HX_) and hot free carriers is determined by *E*_b_ via a modified Saha equation:^113−115^
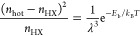
1where λ is the thermal de Broglie wavelength, and *T* is the temperature of the lattice.

The experimental data agree with the predictions of a numerical kinetic model depicted in Figure 5b and described in full detail in SI Section D. The basis of the model is the subject of prior work^95^ and is modified here to account for electron–hole coupling within excitons. According to the model, hot carrier dynamics are represented by the following equation:
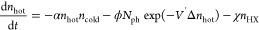
2where α and ϕ are rate coefficients for hot–cold carrier and carrier–phonon scattering, respectively, based on values obtained previously,^95^ and χ is the hot exciton cooling enhancement coefficient obtained from fitting. The total phonon density is represented by *N*_ph_, the carrier–phonon interaction (polaron) volume by *V*′, and the change in hot state density after the push pulse by Δ*n*_hot_. In systems with larger exciton binding energies, a greater proportion of the excited state population exists as excitons (eq 1), increasing the total decay rate of *n*_hot_ (eq 2 and Figure S10). This correlation indicates that carrier–carrier interactions—specifically electron–hole coupling within an exciton—play a dominant role in the cooling dynamics. Therefore, the effect of quantum confinement on hot carrier dynamics is mostly imposed via modification of the electronic states. On the contrary, phonons and carrier–phonon coupling have a secondary role in confined systems and should be considered only in those with low exciton binding energies.

## Conclusions

In summary, we have applied ultrafast PPP spectroscopy to characterize the hot carrier cooling dynamics in LHP nanosystems spanning a range of sizes and shapes. Unlike conventional transient optical methods, the PPP technique allows systematic measurements of the hot phonon bottleneck effect at low (cold) carrier densities. Within this regime, we observe the most pronounced hot phonon bottleneck in three-dimensionally confined cuboidal CsPbBr_3_ NCs, with behavior that closely resembles the bulk material. In these moderately confined systems, the hot phonon bottleneck is weakly dependent on the NC size. However, measurements on 2D systems, including CsPbBr_3_ NPLs and RP-type LHPs, revealed a clear suppression of the hot phonon bottleneck. Based on the developed kinetic model, we propose that carrier–carrier scattering plays a dominant role in the hot carrier photophysics of excitonic systems, especially 2D LHPs. Meanwhile, confinement effects on phonon localization and electron–phonon coupling have a secondary role in carrier cooling dynamics. This could be instructive for tailoring the optical and electronic properties of next-generation LHP-based devices. Finally, the results herein highlight the need to consider carrier density-dependence when comparing hot carrier cooling dynamics across different material systems.

## Methods

### Ultrafast Spectroscopy

A Ti:sapphire regenerative amplifier (Astrella, Coherent, λ_c_ ∼ 800 nm, τ ∼ 35 fs) seeded two optical parametric amplifiers (TOPAS-Prime, Coherent) to produce near-infrared light. The output of “OPA-1” was tuned to ∼1200 nm and coupled into a β-barium borate crystal alongside residual 800 nm light, producing the 490 nm “pump”, which was then modulated at 2 kHz by an optical chopper. The output of “OPA-2” was tuned to 2 μm, where it was then split into two paths by a beamsplitter, with 90% of the incident light forming the “push” and the remaining 10% forming the “probe”. Pump and probe beams were sent into separate mechanical delay stages: the pump–push delay time was controlled through the position of the pump stage, which was fixed for all pump–push–probe measurements at ∼12 ps. The pump–probe delay time was scanned by moving the probe stage. The pump and push fluences were controlled using a neutral density filter wheel. The pump and probe then adopted a collinear geometry and were focused onto a ∼200 μm diameter spot on the sample housed in a N_2_-filled quartz cuvette; the off-axis push was defocused to ∼400 μm to reduce photodegradation and aid spatial overlap. The transmission of the probe was detected by an amplified PbSe photodetector (PDA20H-EC, Thorlabs), and the differential signal was read out by a lock-in amplifier (MFLI, Zurich Instruments).

### CsPbBr_3_ 5 nm NCs

*Chemicals:* Cesium carbonate (Cs_2_CO_3_, Fluorochem), lead(II) acetate trihydrate (99.99%), bromine (99.9%), 1-octadecene (ODE, technical grade), 3-(*N*,*N*-dimethyloctadecylammonio)propanesulfonate (>99%, ASC18), oleic acid (OA, 90%, Sigma-Aldrich/Merck), toluene, acetone (HPLC grade), ethyl acetate (HPLC grade, Fischer), and trioctylphosphine (TOP, >97%, STREM) were obtained.

*Cs-oleate Precursor, 0.4 M in ODE:* Cs_2_CO_3_ (1.628 g, 5 mmol), OA (5 mL, 16 mmol), and ODE (20 mL) were evacuated at 25–120 °C until completion of gas evolution.

*Pb-oleate Precursor, 0.5 M in ODE:* Lead(II) acetate trihydrate (4.607 g, 12 mmol), OA (7.6 mL, 24 mmol), and ODE (16.4 mL) were mixed in a three-necked flask and evacuated at 25–120 °C until complete evaporation of acetic acid and water.

*TOP-Br*_2_*Precursor, 0.5 M in Toluene:* TOP (6 mL, 13 mmol) and bromine (0.6 mL, 11.5 mmol) were mixed under an inert atmosphere. Once the reaction was complete and cooled to room temperature, TOP-Br_2_ was dissolved in toluene (18.7 mL).

*Synthesis of CsPbBr*_3_*NCs with ASC18 Ligand:* CsPbBr_3_ NCs were synthesized by dissolving Cs-oleate (4 mL, 1.6 mmol), Pb-oleate (5 mL, 2.5 mmol), and ASC18 (0.215 g, 0.512 mmol) in ODE (10 mL) and heating the mixture to 130 °C under vacuum, whereupon the atmosphere was changed to argon, and TOP-Br_2_ in toluene (5 mL, 5 mmol Br) was injected. The reaction mixture was cooled immediately in an ice bath.

The crude solution (24 mL) was precipitated by the addition of ethyl acetate (20 mL) and acetone (21 mL) in a nitrogen-filled glovebox, followed by centrifugation at 29500*g* for 10 min. The precipitated fraction was dispersed in toluene (3 mL) and then washed three more times. Each time the solution was mixed with two volumetric equivalents of acetone and centrifuged at 1300*g* for 10 min before being dispersed in progressively smaller volumes of solvent (1.5 mL, then 0.75 mL). After the final precipitation, NCs were dispersed in toluene (3 mL) and centrifuged at 1300*g* for 1 min to remove nondispersed residue.

*CsPbBr*_3_*NC Films:* Z-cut single crystalline quartz substrates were cleaned by sonication in soap water and water stream and then blow-dried (sequence repeated twice). They were then sonicated in ethanol and separately in acetone—blow-drying between steps—and covered with a monolayer of hexamethyldisilazane (HMDS) before annealing in a nitrogen-filled glovebox. Films were prepared by drop-casting from a 0.8 mg/mL NC solution.

### CsPbBr_3_ NPLs

*Chemicals:* Cesium carbonate (Cs_2_CO_3_, Aldrich, 99.9%), oleic acid (OA, Sigma-Aldrich, 90%), 1-octadecene (ODE, Sigma-Aldrich, 90%), oleylamine (OAm, Acros Organics, 80–90%), lead bromide (PbBr_2_, ABCR, 98%), mesitylene (Aldrich, 97%), and toluene (Fischer Scientific, HPLC grade) were obtained. All materials were used without any further purification except OA and OAm, which were predried for 1 h under a vacuum at 125 °C and stored in the glovebox.

*Cs-oleate Precursor:* Cs_2_CO_3_ (0.4075 g), OA (1.25 mL), and ODE (20 mL) were added into a 50 mL three-neck flask and vigorously stirred under a vacuum for 1 h at 120 °C, turning into a transparent slightly yellowish solution. Since Cs-oleate precipitates out of ODE at room temperature, it must be preheated to 100 °C before injection.

*Synthesis of CsPbBr*_3_*NPLs:* PbBr_2_ (138 mg) along with predried OA and OAm were loaded into a 25 mL 3-neck round-bottom flask in the glovebox (N_2_ atmosphere). The flask was transferred to a Schlenk line, and mesitylene (5 mL) was added to the reaction mixture. The system was flushed (N_2_/vac.) three times at room temperature, after which the temperature was raised to 130 °C under N_2_ flow. When 130 °C was reached, 0.8 mL of Cs-oleate prepared as described above was swiftly injected, and the reaction was immediately stopped by immersing the reaction flask into a cold-water bath. After the reaction, 1 mL of crude solution was centrifuged for 3 min at 5000 rpm. The obtained precipitate was dispersed in 1 mL of toluene and centrifuged again for 10 min at 13400 rpm, and the supernatant was filtrated and used for the experiments.

### (PEA)_2_PbI_4_

The precursor solution was prepared by dissolving PEAI (GreatCell Solar) and PbI_2_ (TCI) in a stoichiometric ratio in DMF (Acros) to obtain a 0.25 M solution. Prior to deposition, quartz substrates were sequentially cleaned in acetone, ethanol, and 2-propanol (IPA) by ultrasonication for 10 min, followed by an oxygen plasma treatment at 100 mW for 10 min. The drop-cast films were prepared by dropping 100 μL of the solution on a substrate to cover it completely, followed by 10 min of annealing at 100 °C. A home-built setup was used for fabrication of zone-cast films. The substrates were placed on a 100 °C hot aluminum block and moved slowly (0.02 mm/s) using stepper motors below a nozzle, which supplied a meniscus of the perovskite solution.

### (PEA)_2_PbBr_4_

500 μL of 0.5 M PbBr_2_ (TCI) solution in DMF was mixed with 1000 μL of 0.5 M PEABr (GreatCell) solution in DMF and stirred at 70 °C for 10 min. The films were then deposited by spin-coating for 30 s at 4000 rpm and subsequent annealing at 100 °C for 10 min.

## Supporting Information Available

Effect of PPP IRF; TEM and XRD characterization data; PPP data for (PEA)_2_PbBr_4_; further descriptions of material synthesis and ultrafast spectroscopy experiments; and details of the Monte Carlo, Elliott, and kinetic models (PDF)
