# Supplementary material for: Confinement and Exciton Binding Energy Effects on Hot Carrier Cooling in Lead Halide Perovskite Nanomaterials
Source: ACS Nano. 2023 Mar 20;17(7):6638–48. doi: 10.1021/acsnano.2c12373 (PMC10100565; doi:10.1021/acsnano.2c12373)
Supplement: Supplementary file 1 — nn2c12373_si_001.pdf [file nn2c12373_si_001.pdf]

Supporting Information

for

Confinement and Exciton Binding Energy Effects on Hot Carrier Cooling in Lead Halide Perovskite Nanomaterials

Ben P. Carwithen[1], Thomas R. Hopper[1,2], Ziyuan Ge[1], Navendu Mondal[1], Tong Wang[1], Rozana Mazlumian[1], Xijia Zheng[1], Franziska Krieg[3,4], Federico Montanarella[3,4], Georgian Nedelcu[3,4,5], Martin Kroll[6,7], Miguel Albaladejo Siguan[8,9], Jarvist M. Frost[1], Karl Leo[7], Yana Vaynzof[8,9], Maryna I. Bodnarchuk[3,4], Maksym V. Kovalenko[3,4], Artem A. Bakulin[1]*

*Email: a.bakulin@imperial.ac.uk

[1]*Department of Chemistry and Centre for Processable Electronics, Imperial College London, W12 0BZ, United Kingdom*

[2]*Department of Materials Science and Engineering, Stanford University, Stanford, CA 94305, USA*

[3]*Laboratory of Inorganic Chemistry, Department of Chemistry and Applied Biosciences, ETH Zürich, CH-8093 Zürich, Switzerland*

[4]*Laboratory for Thin Films and Photovoltaics, Empa − Swiss Federal Laboratories for Materials Science and Technology, CH-8600 Dübendorf, Switzerland*

[5]*Zernike Institute for Advanced Materials, University of Groningen, Nijenborgh 4, Groningen, 9747AG, The Netherlands*

[6]*Center for Advancing Electronics Dresden, Technische Universität Dresden, 01069 Dresden, Germany*

[7]*Integrated Center for Applied Photophysics and Photonic Materials, Technische Universität Dresden, 01187 Dresden, Germany*

[8]*Chair for Emerging Electronic Technologies, Technische Universität Dresden, 01187 Dresden, Germany*

[9]*Leibniz Institute for Solid State and Materials Research Dresden, Technische Universität Dresden, 01069 Dresden, Germany*

Keywords: hot carriers, two-dimensional perovskites, nanoplatelets, nanocrystals, ultrafast spectroscopy

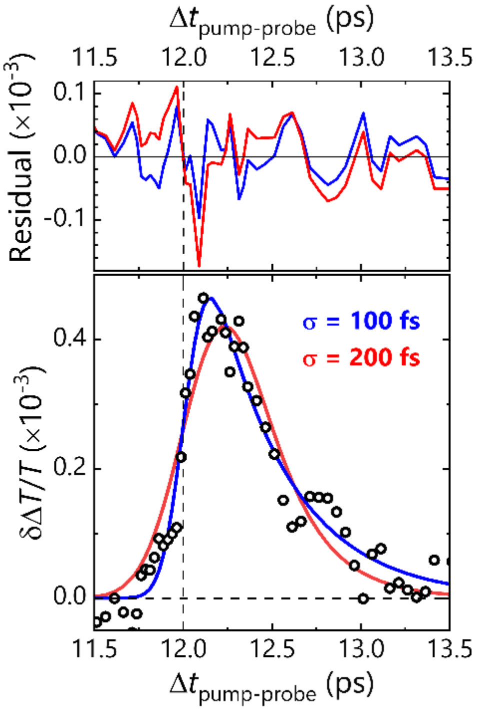

Figure S1: *To extract the cooling time constants from the push-induced PPP dynamics, all data are fitted with a convolution of a Gaussian and exponential function. The bleach rise is most accurately modelled when σ = 100 fs and is used for all data sets.*

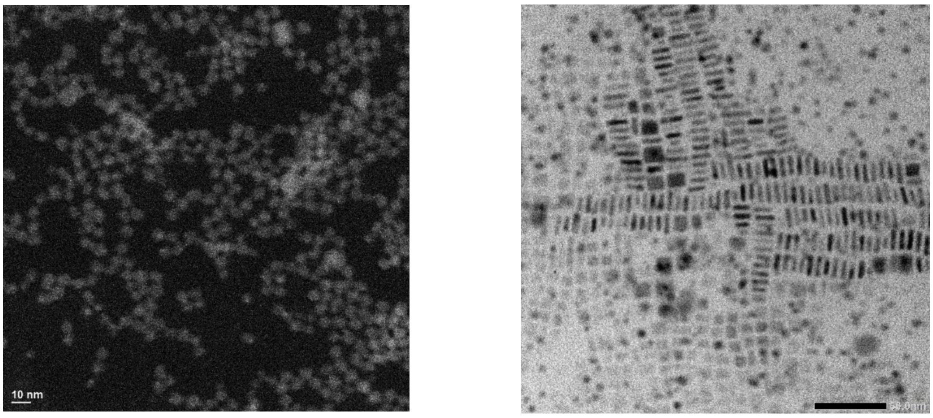

Figure S2: (Left) *Scanning tunnelling electron micrograph of 5 nm $CsPbBr_3$ NCs, scale bar = 10 nm;* ***(Right)*** *Transmission electron micrograph of 3.5 nm $CsPbBr_3$ NPLs, scale bar = 50 nm.*

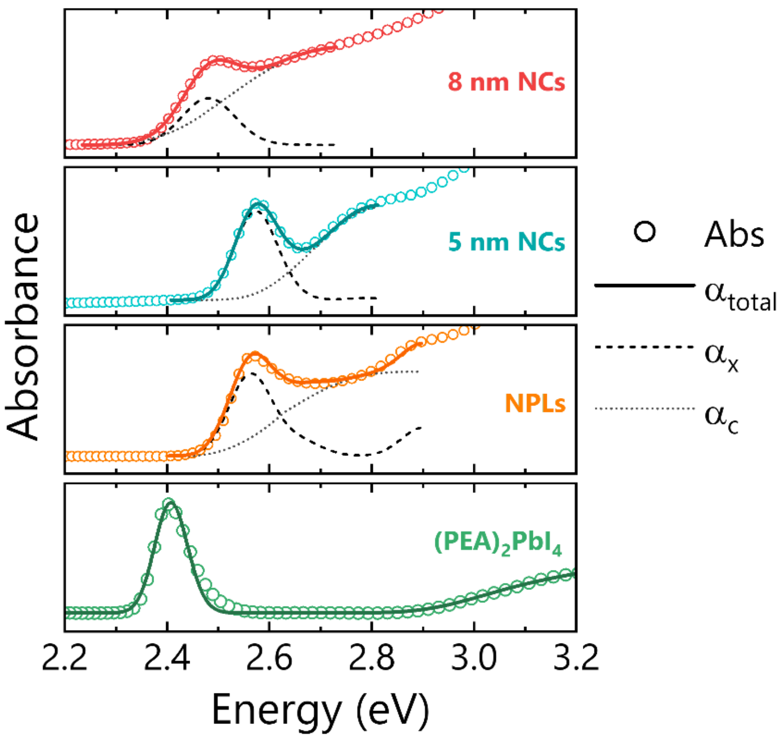

Figure S3: *The absorption spectra of the studied materials were modelled using Elliott theory described in Section C below. The more confined systems display a larger exciton binding energy and further excitonic transitions above the band gap.*

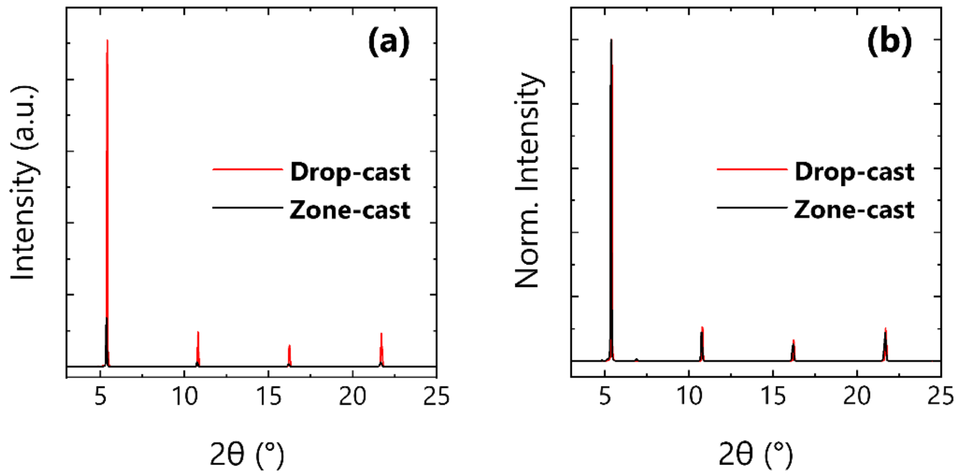

Figure S4: *X-ray diffraction patterns for drop- and zone-cast* $(PEA)_2PbI_4$ *films. (a) Absolute counts; (b) intensities normalised to largest peak in respective sample.*

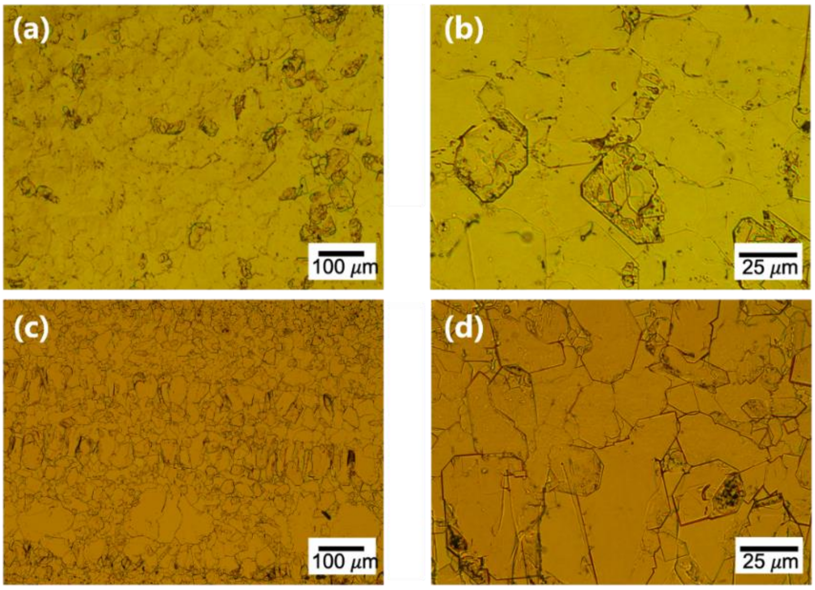

Figure S5: *Optical microscope images of (a,b) drop-cast and (c,d) zone-cast* $(PEA)_2PbI_4$ *films.*

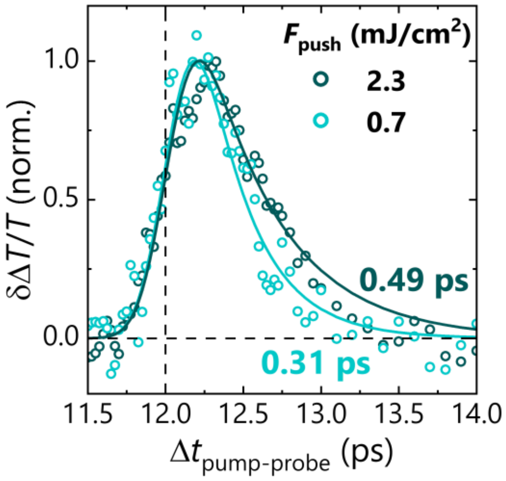

Figure S6: *Normalised PPP bleach in 5 nm* $CsPbBr_3$ *NCs. The bleach recovery slows at higher push fluence due to the hot phonon bottleneck. Cooling time constants are 0.49±0.03 and 0.31±0.02 ps.*

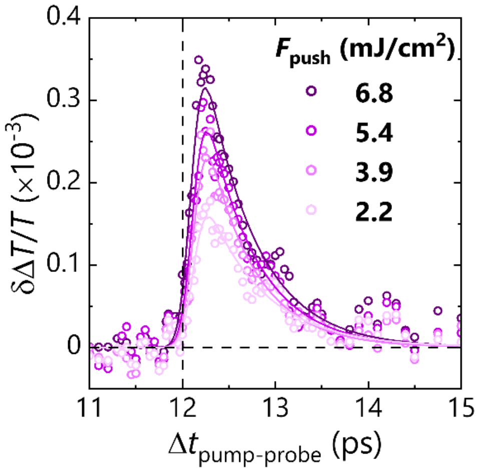

Figure S7: *Exemplar push fluence-dependent PPP bleach kinetics for drop-cast (PEA)$_2$PbI$_4$ at a fixed pump fluence of 30 μJ cm^{-2}.*

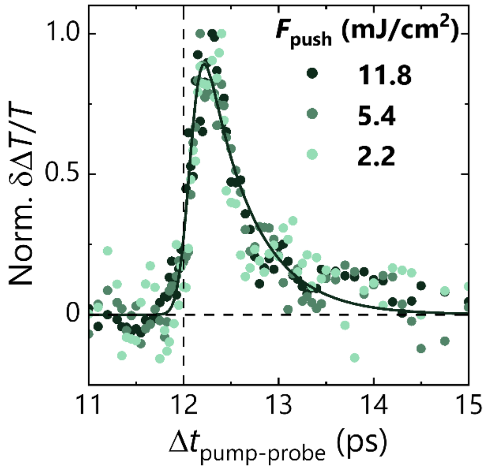

Figure S8: *Normalised PPP bleach in zone-cast (PEA)$_2$PbI$_4$, displaying negligible dependence on push fluence (or hot carrier density).*

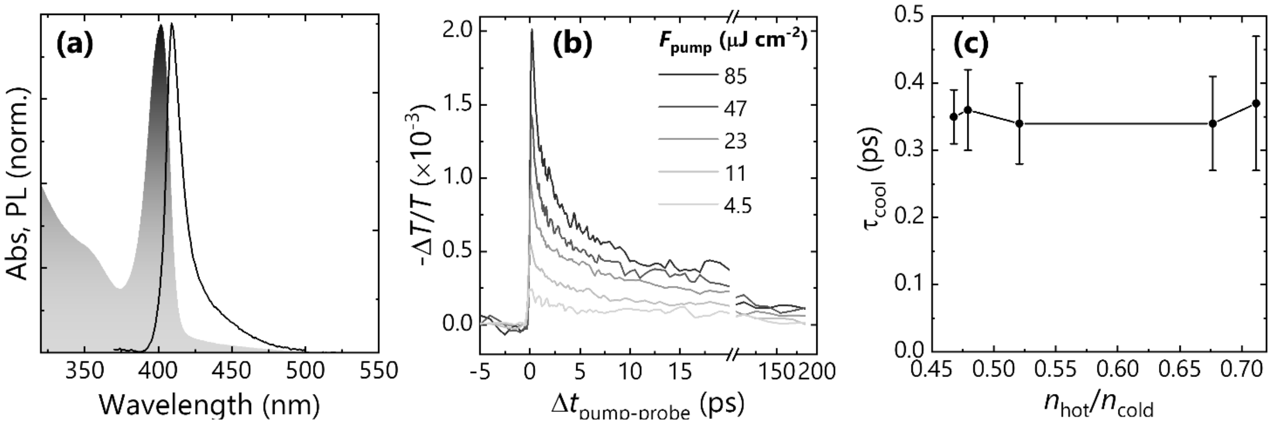

Figure S9: *(a) Absorbance and PL spectra for $(PEA)_2PbBr_4$; (b) Pump fluence-dependent kinetics of the intraband excited state absorption (λ_{pump}=400 nm; λ_{push} = 1300 nm; λ_{probe} = 1300 nm); (c) The hot carrier lifetime shows no dependence on hot carrier density, which is consistent with the suppressed hot phonon bottleneck also observed in $(PEA)_2PbI_4$.*

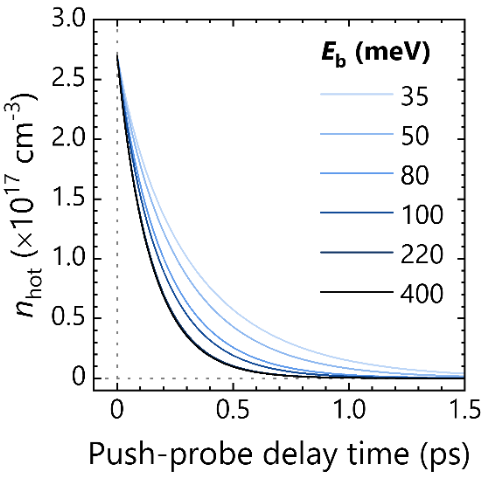

Figure S10: *Hot carrier dynamics simulated using the kinetic model described in Section D below. Hot carrier cooling is faster in systems with higher exciton binding energy.*

A. Materials and methods

$CsPbBr_3$ 5 nm NCs

Chemicals: Cesium carbonate (Cs_2CO_3, Fluorochem), lead (II) acetate trihydrate (99.99%), bromine (99.9%), 1-octadecene (ODE, technical grade), 3-(N,N-dimethyloctadecylammonio)propanesulfonate (>99% , ASC18) and oleic acid (OA, 90%, Sigma Aldrich/Merck), toluene, acetone (HPLC grade), ethylacetate (HPLC grade, Fischer), trioctylphosphine (TOP, >97%, STREM).

Cs-oleate precursor, 0.4 M in ODE: Cs_2CO_3 (1.628 g, 5 mmol), OA (5 ml, 16 mmol) and ODE (20 ml) were evacuated at 25-120 °C until completion of gas evolution.

Pb-oleate precursor, 0.5 M in ODE: lead (II) acetate trihydrate (4.607 g, 12 mmol), OA (7.6 ml, 24 mmol) and ODE (16.4 ml) were mixed in a three-necked flask and evacuated at 25-120 °C until complete evaporation of acetic acid and water.

TOP-Br_2 precursor, 0.5 M in toluene: TOP (6 ml, 13 mmol) and bromine (0.6 ml, 11.5 mmol) were mixed under inert atmosphere. Once the reaction was complete and cooled to room temperature, the TOP-Br_2 was dissolved in toluene (18.7 ml).

Synthesis of $CsPbBr_3$ NCs with ASC18 ligand: $CsPbBr_3$ NCs were synthesised by dissolving Cs-oleate (4 ml, 1.6 mmol), Pb-oleate (5 ml, 2.5 mmol) and ASC18 (0.215 g, 0.512 mmol) in ODE (10 ml) and heating the mixture to 130 °C under vacuum, whereupon the atmosphere was changed to argon and TOP-Br_2 in toluene (5 ml, 5 mmol Br) was injected. The reaction was cooled immediately in an ice bath.

The crude solution (24 ml) was precipitated by the addition of ethyl acetate (20 ml) and acetone (21 ml) in a nitrogen-filled glovebox, followed by centrifugation at 29500*g* for 10 min. The precipitated fraction was dispersed in toluene (3 ml) and then washed three more times. Each time the solution was mixed with two volumetric equivalents of acetone and centrifuged at 1300*g* for 10 min, before being dispersed in progressively smaller volumes of solvent (1.5 ml, then 0.75 ml). After the final precipitation, NCs were dispersed in toluene (3 ml) and centrifuged at 1300*g* for 1 min to remove non-dispersed residue.

$CsPbBr_3$ NC films: Z-cut single crystalline quartz substrates were cleaned by sonication in soap water and water stream, then blow dried (sequence repeated twice). They were then sonicated in ethanol, and separately in acetone – blow-drying between steps – and covered with a monolayer of hexamethyldisilazane before annealing in a nitrogen-filled glovebox. Films were prepared by drop-casting a 0.8 mg/ml NC solution.

$CsPbBr_3$ NPLs

Chemicals: Cesium carbonate (Cs_2CO_3, Aldrich, 99.9%), oleic acid (OA, Sigma-Aldrich, 90%), 1-octadecene (ODE, Sigma-Aldrich, 90%), oleylamine (OAm, Acros Organics, 80–90%), lead bromide ($PbBr_2$, ABCR, 98%), mesitylene (Aldrich, 97%), toluene (Fischer Scientific, HPLC grade). All materials were used without any further purification except OA and OAm, which were pre-dried for 1 h under vacuum at 125 °C and stored in the glovebox.

Cs-oleate precursor: Cs_2CO_3 (0.4075 g), OA (1.25 ml), and ODE (20 ml) were added into a 50 ml three-neck flask and vigorously stirred under vacuum for 1 h at 120 °C, turning into a transparent slightly yellowish solution. Since Cs-oleate precipitates out of ODE at room temperature, it must be preheated to 100 °C before injection.

Synthesis of $CsPbBr_3$ NPLs: $PbBr_2$ (138 mg) along with pre-dried OA and OAm were loaded into a 25 ml 3-neck round-bottom flask in the glovebox (N_2 atmosphere). The flask was transferred to a Schlenk line and mesitylene (5 ml) were added to the reaction mixture. The system was flushed (N_2/vac.) three times at room temperature, after which the temperature was raised to 130 °C under N_2 flow. When 130 °C was reached, 0.8 ml Cs-oleate prepared as described above was swiftly injected and the reaction was immediately stopped by immersing the reaction flask into a cold-water bath. After the reaction, 1 ml of crude solution was centrifuged for 3 minutes at 5000 rpm. The obtained precipitate was dispersed in 1 ml toluene, centrifuged again for 10 minutes at 13400 rpm, and the supernatant was filtrated and used for the experiments.

$(PEA)_2PbI_4$

The precursor solution was prepared by dissolving PEAI (GreatCell Solar) and PbI_2 (TCI) in stoichiometric ratio in DMF (Acros) to obtain a 0.25 M solution. Prior to deposition, quartz substrates were sequentially cleaned in acetone, ethanol and 2-propanol (IPA) by ultrasonication for 10 min, followed by an oxygen plasma treatment at 100 mW for 10 min. The drop-cast films were prepared by dropping 100 µl of the solution on a substrate to cover it completely, followed by 10 min annealing at 100 °C. A home-built setup was used for fabrication of zone-cast films. The substrates were placed on a 100 °C hot aluminium block and moved slowly (0.02 mm/s) using stepper motors below a nozzle, which supplied a meniscus of the perovskite solution.

$(PEA)_2PbBr_4$

500 µl of 0.5 M $PbBr_2$ (TCI) solution in DMF was mixed with 1000 µl of 0.5 M PEABr (GreatCell) solution in DMF and stirred at 70 °C for 10 min. The films were then deposited by spin-coating for 30 s at 4000 rpm and subsequent annealing at 100 °C for 10 minutes.

Ultrafast spectroscopy

A Ti:sapphire regenerative amplifier (Astrella, Coherent, $\lambda_c \sim 800$ nm, $\tau \sim 35$ fs) seeded two optical parametric amplifiers (TOPAS-Prime, Coherent) to produce near-infrared light. The output of "OPA-1" was tuned to ~1200 nm and coupled into a β-barium borate crystal alongside residual 800 nm light, producing the 490 nm "pump", which was then modulated at 2 kHz by an optical chopper. The output of "OPA-2" was tuned to 2 µm, where it was then split into two paths by a beamsplitter, with 90% of the incident light forming the "push" and the remaining 10% the "probe". Pump and probe beams were sent into separate mechanical delay stages: the pump-push delay time was controlled through the position of the pump stage, which was fixed for all pump-push-probe measurements at ~12 ps. The pump-probe delay time was scanned by moving the probe stage. The pump and push fluences were controlled using a neutral density filter wheel. The pump and probe then adopted a collinear geometry and were focused onto a ~200 µm diameter spot on the sample housed in a N_2-filled quartz cuvette; the off-axis push was defocused to ~400 µm to reduce photodegradation and aid spatial overlap. The transmission of the probe was detected by an amplified PbSe photodetector (PDA20H-EC, Thorlabs), and the differential signal read out by a lock-in amplifier (MFLI, Zurich Instruments).

B. Monte Carlo simulation for cooling within NCs

To demonstrate the validity of assuming an effectively bulk-like carrier density throughout the studied systems, we have modelled the push pulse action on a test set of confined NCs using a Monte Carlo algorithm. Here, $N_{h\nu}$ push photons are randomly assigned to 10000 NCs. With increasing $N_{h\nu}$, the probability distribution of forming 0, 1 or 2 hot carriers in each NC shifts to higher values. If we assume that cooling within a singly occupied NC takes τ_1, and cooling in a doubly occupied NC takes τ_2 (where $\tau_1 < \tau_2$ due to the hot phonon bottleneck), then the weighted average cooling time across all NCs increases smoothly with $N_{h\nu}$. The following result was obtained with τ_1 = 200 fs and τ_2 = 600 fs – these values are arbitrarily chosen but the same trend holds provided $\tau_1 < \tau_2$.

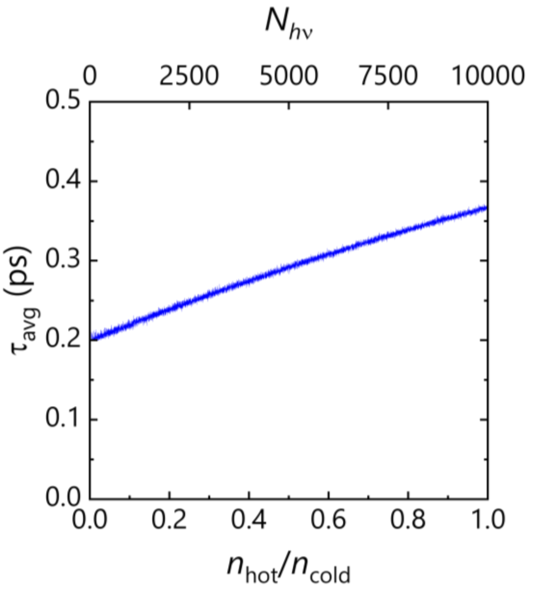

Figure S11: *Average cooling time increases smoothly with hot carrier density when modelled in entirely confined systems. The same behaviour is seen for bulk free carrier systems which further validates the choice of* $n_{\text{hot}}/n_{\text{cold}}$ *as a metric to compare all systems presented.*

This result obtained from a strictly confined system is very similar to that predicted by a bulk free carrier model.[1,2] As such, both classes of system and their intermediates can be described by the same model as used in the manuscript.

C. Fitting of absorption spectra with Elliott model

To verify the exciton binding energy of each studied material, we apply an Elliott model to the absorption spectra, which are decomposed into a linear combination of contributions by exciton (α_x) and continuum (α_c) states:

$$\alpha(E) = \alpha_\mathrm{x}(E) + \alpha_\mathrm{c}(E) \tag{1}$$

$$\alpha_\mathrm{x}(E) = \left[A_\mathrm{x}\sum_{n=1}^{\infty}\frac{1}{n^3}\cdot\delta\left(E - E_\mathrm{g} + \frac{E_\mathrm{b}}{n^2}\right)\right] + \left[B_\mathrm{x}\cdot\delta(E - E_2)\right] \tag{2}$$

$$\alpha_\mathrm{c}(E) = A_\mathrm{c}\cdot\theta\left(\sqrt{E - E_\mathrm{g}}\right), \qquad \theta = \begin{cases}\sqrt{E - E_\mathrm{g}}, & E > E_\mathrm{g}\\ 0, & E \leq E_\mathrm{g}\end{cases} \tag{3}$$

The sub-gap exciton states are modelled in the first term of (2) by a series of delta functions at $E = E_\mathrm{g} - \frac{E_\mathrm{b}}{n^2}$ (so converges at E_g), with intensities that scale with $1/n^3$. In practice, $n \lesssim 5$ is typically sufficient. The second term of (2) describes an exciton-like transition found above the band gap at $E = E_2$ in the more confined systems.

Above E_g, the continuum scales with $\sqrt{E - E_\mathrm{g}}$, which assumes a parabolic band structure near the band edge.

A_x, B_x and A_c are scaling factors to fit the model to the experimental data.

Line broadening due to static and dynamic disorder is modelled by convolving the exciton and continuum contributions with Gaussian functions. The final fitted spectra thus have the form:

$$\alpha_\mathrm{total}(E) = \alpha_\mathrm{x}(E)\otimes G(E,\sigma_\mathrm{x}) + \alpha_\mathrm{c}(E)\otimes G(E,\sigma_\mathrm{c}) \tag{4}$$

The results for each material are plotted in Figure S2 and show good agreement with the absorption spectra using E_b values similar to those reported previously.[3–6] It has been shown that the absorption profile of bulk perovskites at the band edge is sensitive to fabrication procedures which cause exciton features to be more pronounced despite a relatively low E_b in these systems.[7] The value used is therefore based on the literature and not verified further in this work. In the case of the $(PEA)_2PbI_4$ absorption

spectrum, subtraction of a spline describing the profile between the exciton and continuum contributions yields a better fit to the model.

D. Kinetic model for the effect of exciton binding energy on hot carrier cooling dynamics

To gain further insight into the effect of the materials' excitonic character on their hot carrier cooling dynamics, we have developed a numerical kinetic model based on a system of coupled differential equations:

$$\frac{\mathrm{d}n_{\mathrm{hot}}}{\mathrm{d}t} = -\alpha n_{\mathrm{hot}} n_{\mathrm{cold}} - \phi n_{\mathrm{hot}} n_{\mathrm{ph}} - \chi n_{\mathrm{HX}} \tag{1}$$

$$\frac{\mathrm{d}n_{\mathrm{cold}}}{\mathrm{d}t} = \alpha n_{\mathrm{cold}} n_{\mathrm{hot}} + \phi n_{\mathrm{ph}} n_{\mathrm{hot}} + \chi n_{\mathrm{HX}} \tag{2}$$

$$\frac{\mathrm{d}n_{\mathrm{ph}}}{\mathrm{d}t} = -\phi n_{\mathrm{ph}} n_{\mathrm{hot}} \tag{3}$$

These rate equations are based on the following assumptions:

- $n_{\mathrm{hot}} = n_{\mathrm{HC}} + n_{\mathrm{HX}}$, where n_{HC} is the hot (free) carrier density and n_{HX} is the hot exciton density;
- Excited states may be either hot or cold;
- HCs and HXs become cold with identical efficiencies via two mechanisms: (i) energy deposition into vacant phonons (with density n_{ph}); and (ii) scattering with the reservoir of cold states (with density n_{cold});
- An additional cooling pathway is available to the HXs;
- Occupied phonon modes may not contribute to cooling;
- α, ϕ and χ are the rate constants for hot-cold carrier (or carrier-exciton, exciton-exciton) scattering, carrier-phonon scattering and hot exciton cooling, respectively;
- Band-to-band recombination and occupied phonon freeing rates are negligible on timescales relevant for hot carrier cooling.

We note that under relatively soft assumptions, equation (3) can be excluded and equation (1) takes the form:

$$\frac{\mathrm{d}n_{\mathrm{hot}}}{\mathrm{d}t} = -\alpha n_{\mathrm{hot}} n_{\mathrm{cold}} - \phi N_{\mathrm{ph}} \exp(-V' \Delta n_{\mathrm{hot}}) - \chi n_{\mathrm{HX}} \tag{1*}$$

The total phonon density is represented by N_{ph}, the carrier-phonon interaction (polaron) volume by V', and Δn_{hot} is the change in hot state density after the push pulse. This equation is given in the main text of the paper.

The following initial conditions are imposed for the simulations:

- $n_{\mathrm{hot}}(0) = I_{\mathrm{push}} \times n_{\mathrm{pump}}$;
- $n_{\mathrm{cold}}(0) = (1 - I_{\mathrm{push}}) \times n_{\mathrm{pump}}$; where $0 < I_{\mathrm{push}} < 1$ and describes the intensity of the push pulse and thus the optically re-excited portion of the total (cold) excited state density formed by the pump (n_{pump}). The push is modelled as being instantaneous for simplicity;
- $n_{\mathrm{ph}}(0) = N_{\mathrm{ph}}$, the total number of phonon modes, i.e. all phonon modes are assumed empty at the instant hot carriers are formed by the push.

The hot carrier-exciton partition is based on a modified Saha equation:[8,9]

$$\frac{x^2}{1-x} = \frac{1}{n_{\mathrm{hot}} \lambda^3} e^{-(E_{\mathrm{b}}/k_{\mathrm{B}}T)} \tag{4}$$

where $x = n_{\mathrm{HC}}/n_{\mathrm{hot}}$, $\lambda = h/\sqrt{2\pi\mu k_{\mathrm{B}}T}$, E_{b} is the material's exciton binding energy, and T is temperature. We assume a reduced electron-hole mass of $\mu = 0.2m_{\mathrm{e}}$ for all materials, and do not attempt to account for polaron or trap effects here.[10,11]

The system of rate equations can then be expressed as:

$$\frac{\mathrm{d}n_{\mathrm{hot}}}{\mathrm{d}t} = -\alpha n_{\mathrm{hot}} n_{\mathrm{cold}} - \phi n_{\mathrm{hot}} n_{\mathrm{ph}} - \chi(1-x) n_{\mathrm{hot}} \tag{5}$$

$$\frac{\mathrm{d}n_{\mathrm{cold}}}{\mathrm{d}t} = \alpha n_{\mathrm{cold}} n_{\mathrm{hot}} + \phi n_{\mathrm{ph}} n_{\mathrm{hot}} + \chi(1-x) n_{\mathrm{hot}} \tag{6}$$

$$\frac{\mathrm{d}n_{\mathrm{ph}}}{\mathrm{d}t} = -\phi n_{\mathrm{ph}} n_{\mathrm{hot}} \tag{7}$$

where x is derived by rearranging (4) and substituting $\xi = E_{\mathrm{b}}/k_{\mathrm{B}}T$ to give:

$$x^2 + \frac{e^{-\xi}}{n_{\mathrm{hot}}\lambda^3} x - \frac{e^{-\xi}}{n_{\mathrm{hot}}\lambda^3} = 0 \tag{8}$$

$$x = -\frac{e^{-\xi}}{2n_{\mathrm{hot}}\lambda^3} \pm \frac{1}{2}\sqrt{\left(\frac{e^{-\xi}}{n_{\mathrm{hot}}\lambda^3}\right)^2 + \frac{4e^{-\xi}}{n_{\mathrm{hot}}\lambda^3}} \tag{9}$$

Note that the quadratic equation in (9) has exactly one positive real solution, which is taken as the true value of x.

The Euler method is employed to approximate the solutions to the differential equations and simulate the time-dependent population dynamics. For example, the change in n_{hot} from a time point $(i-1)$ to the next (i) is described by:

$$n_{\mathrm{hot}}(t_i) = n_{\mathrm{hot}}(t_{i-1}) + \frac{\mathrm{d}n_{\mathrm{hot}}(t_{i-1})}{\mathrm{d}t} \Delta t_{i-1\to i} \tag{10}$$

That is, the change in n_{hot} between any two time points is linear, which is a reasonable approximation for sufficiently small time steps. τ_{cool} is extracted by fitting the simulated n_{hot} dynamics with an exponential function of the form $f(t) \propto e^{-t/\tau_{\mathrm{cool}}}$.

The dependence (β in the main text) of the simulated cooling time constant (τ_{cool}) on push intensity (I) is evaluated for a range of E_{b} values, and shows an increasingly suppressed hot phonon bottleneck for more excitonic systems. The model is in good agreement with experimental results using the following parameters:

α = 1.0×10^{-18} cm^3 ps^{-1}, ϕ = 1.8×10^{-17} cm^3 ps^{-1}, χ = 5.1 ps^{-1}, N_{ph} = 1×10^{17} cm^{-3}, n_{pump} = 3×10^{17} cm^{-3}.

References

(1) Hopper, T. R.; Jeong, A.; Gorodetsky, A. A.; Krieg, F.; Bodnarchuk, M. I.; Huang, X.; Lovrincic, R.; Kovalenko, M. V.; Bakulin, A. A. Kinetic Modelling of Intraband Carrier Relaxation in Bulk and Nanocrystalline Lead-Halide Perovskites. *Phys. Chem. Chem. Phys.* **2020**, *22*, 17605–17611.

(2) Hopper, T. R.; Gorodetsky, A.; Jeong, A.; Krieg, F.; Bodnarchuk, M. I.; Maimaris, M.; Chaplain, M.; Macdonald, T. J.; Huang, X.; Lovrincic, R.; Kovalenko, M. V.; Bakulin, A. A. Hot Carrier Dynamics in Perovskite Nanocrystal Solids: Role of the Cold Carriers, Nanoconfinement, and the Surface. *Nano Lett.* **2020**, *20*, 2271–2278.

(3) Hong, X.; Ishihara, T.; Nurmikko, A. V. Dielectric Confinement Effect on Excitons in PbI_4-Based Layered Semiconductors. *Phys. Rev. B* **1992**, *45*, 6961–6964.

(4) Yang, Z.; Surrente, A.; Galkowski, K.; Miyata, A.; Portugall, O.; Sutton, R. J.; Haghighirad, A. A.; Snaith, H. J.; Maude, D. K.; Plochocka, P.; Nicholas, R. J. Impact of the Halide Cage on the Electronic Properties of Fully Inorganic Cesium Lead Halide Perovskites. *ACS Energy Lett.* **2017**, *2*, 1621–1627.

(5) Li, J.; Yuan, X.; Jing, P.; Li, J.; Wei, M.; Hua, J.; Zhao, J.; Tian, L. Temperature-Dependent Photoluminescence of Inorganic Perovskite Nanocrystal Films. *RSC Adv.* **2016**, *6*, 78311–78316.

(6) Li, J.; Luo, L.; Huang, H.; Ma, C.; Ye, Z.; Zeng, J.; He, H. 2D Behaviors of Excitons in Cesium Lead Halide Perovskite Nanoplatelets. *J. Phys. Chem. Lett.* **2017**, *8*, 1161–1168.

(7) Grancini, G.; Srimath Kandada, A. R.; Frost, J. M.; Barker, A. J.; De Bastiani, M.; Gandini, M.; Marras, S.; Lanzani, G.; Walsh, A.; Petrozza, A. Role of Microstructure in the Electron–Hole Interaction of Hybrid Lead Halide Perovskites. *Nat. Photonics* **2015**, *9*, 695–701.

(8) Saha, M. N. LIII. Ionization in the Solar Chromosphere. *London, Edinburgh, Dublin Philos. Mag. J. Sci.* **1920**, *40*, 472–488.

(9) D'Innocenzo, V.; Grancini, G.; Alcocer, M. J. P.; Kandada, A. R. S.; Stranks, S. D.; Lee, M. M.; Lanzani, G.; Snaith, H. J.; Petrozza, A. Excitons versus Free Charges in Organo-Lead Tri-Halide Perovskites. *Nat. Commun.* **2014**, *5*, 3586.

(10) Simbula, A.; Pau, R.; Wang, Q.; Liu, F.; Sarritzu, V.; Lai, S.; Lodde, M.; Mattana, F.; Mula, G.; Geddo Lehmann, A.; Spanopoulos, I. D.; Kanatzidis, M. G.; Marongiu, D.; Quochi, F.; Saba, M.; Mura, A.; Bongiovanni, G. Polaron Plasma in Equilibrium with Bright Excitons in 2D and 3D Hybrid Perovskites. *Adv. Opt. Mater.* **2021**, *9*, 2100295.

(11) Mariano, F.; Cretì, A.; Carbone, L.; Genco, A.; D'Agostino, S.; Carallo, S.; Montagna, G.; Lomascolo, M.; Mazzeo, M. The Enhancement of Excitonic Emission Crossing Saha Equilibrium in Trap Passivated $CH_3NH_3PbBr_3$ Perovskite. *Commun. Phys.* **2020**, *3*, 41.
